# Supplementary material for: Health care utilization, mental disorders and behavioural disorders among perpetrators of intimate partner homicide in 2000–2016: A registry-based case-control study from Sweden
Source: PLoS One. 2024 Feb 23;19(2):e0298693. doi: 10.1371/journal.pone.0298693 (PMC10889610; doi:10.1371/journal.pone.0298693)
Supplement: Attachment — Submitted filename: Response to reviewers.docx [file pone.0298693.s001.docx]

**Journal requirements:**

When submitting your revision, we need you to address these additional requirements.

1. Please ensure that your manuscript meets PLOS ONE's style requirements, including those for file naming. The PLOS ONE style templates can be found at 
<https://journals.plos.org/plosone/s/file?id=wjVg/PLOSOne_formatting_sample_main_body.pdf> and 
<https://journals.plos.org/plosone/s/file?id=ba62/PLOSOne_formatting_sample_title_authors_affiliations.pdf>

**Response to editor**

**Editor´s comments:**
2. Please provide additional details regarding participant consent. In the ethics statement in the Methods and online submission information, please ensure that you have specified (1) whether consent was informed and (2) what type you obtained (for instance, written or verbal, and if verbal, how it was documented and witnessed). If your study included minors, state whether you obtained consent from parents or guardians. If the need for consent was waived by the ethics committee, please include this information.

**Our comments:**
We have added information about the informed consent in the ethics section of the manuscript, i.e. according to the Regional Ethical Review Board, informed consent is not required for registry-based research.

**Editor´s comments:**
If you are reporting a retrospective study of medical records or archived samples, please ensure that you have discussed whether all data were fully anonymized before you accessed them and/or whether the IRB or ethics committee waived the requirement for informed consent. If patients provided informed written consent to have data from their medical records used in research, please include this information.

**Our comments:**
We have added information about the anonymisation of the data in the ethics section of the manuscript.

**Editor´s comments:**
3. In your Data Availability statement, you have not specified where the minimal data set underlying the results described in your manuscript can be found. PLOS defines a study's minimal data set as the underlying data used to reach the conclusions drawn in the manuscript and any additional data required to replicate the reported study findings in their entirety. All PLOS journals require that the minimal data set be made fully available. For more information about our data policy, please see <http://journals.plos.org/plosone/s/data-availability>.

**Our comment:**
Please see our comments further down in this letter.

""Upon re-submitting your revised manuscript, please upload your study’s minimal underlying data set as either Supporting Information files or to a stable, public repository and include the relevant URLs, DOIs, or accession numbers within your revised cover letter. For a list of acceptable repositories, please see <http://journals.plos.org/plosone/s/data-availability#loc-recommended-repositories>. Any potentially identifying patient information must be fully anonymized.

Important: If there are ethical or legal restrictions to sharing your data publicly, please explain these restrictions in detail. Please see our guidelines for more information on what we consider unacceptable restrictions to publicly sharing data: <http://journals.plos.org/plosone/s/data-availability#loc-unacceptable-data-access-restrictions>. Note that it is not acceptable for the authors to be the sole named individuals responsible for ensuring data access. We will update your Data Availability statement to reflect the information you provide in your cover letter.

**Our comment:**
We apologise that our previous statement under the "Data Availability Statement" section was not sufficiently clear. We would like to clarify the previous statement by replacing it with the following statement:

*“This study is based on data available from the Swedish NBHW and Statistics Sweden. Data were used under license for the current study and cannot be shared publicly due to restrictions stipulated by the registry holders and Swedish Ethical Review Authority. Data are available from The Västra Götaland Region Competence Centre on Intimate Partner Violence (contact via Jenny Ström:* [*jenny.strom@vgregion.se*](mailto:jenny.strom@vgregion.se)*, Tel +46 768-40 22 96) upon reasonable request and with permission of the Swedish NBHW, Statistics Sweden and the Swedish Ethical Review Authority”.*

**Editor´s comments:**
4. We note that you have stated that you will provide repository information for your data at acceptance. Should your manuscript be accepted for publication, we will hold it until you provide the relevant accession numbers or DOIs necessary to access your data. If you wish to make changes to your Data Availability statement, please describe these changes in your cover letter and we will update your Data Availability statement to reflect the information you provide.

**Our comment:**
Please see the revised Data Availability Statement mentioned in our comments under point 3 above.

**Editor´s comments:**
5. Your ethics statement should only appear in the Methods section of your manuscript. If your ethics statement is written in any section besides the Methods, please move it to the Methods section and delete it from any other section. Please ensure that your ethics statement is included in your manuscript, as the ethics statement entered into the online submission form will not be published alongside your manuscript.

**Our comments:**
We have removed ethics statements from the “Study design and study population” section in the manuscript and moved it to the “Ethical considerations” section instead.

Additional Editor Comments:

Dear authors, I kindly request that you take into consideration all the comments from the reviewers. I believe they are relevant and appropriate.

**Response to reviewer # 1**

**Reviewer’s comments:** 
Thank you for the opportunity to review this important work. The manuscript is well-written and compelling. However, it would benefit from some minor adjustments, as follows;
Abstract: The first two sentences are quite repetitive, I would consider revising.

**Our comments**:
Thank you very much for your valuable comments and suggestions for improvement of this paper. We have made changes in the two first sentences in the abstract according to the reviewer’s suggestion: “*Little is known about intimate partner homicide (IPH) perpetrator´s healthcare contacts and mental health problems before the killing. The aim was to compare male and female IPH perpetrators with matched controls from the general population by analysing differences in healthcare utilization and mental and behavioural disorders”.*

We hope this has improved the text, and made the first two sentences less repetitive.

**Reviewer’s comments:**
Methods: There is no information about how the sample was matched, it would be good to know how respondents were chosen and the methods that were used to match them to the perpetrators. It is also important to include any power analysis that helped to calculate the sample size for the study since the sample is so small.

**Our comments:**
In the methods section, we explain how the controls were matched, i.e. for each offender, 10 controls were randomly selected and matched for sex, birth year and residential area at the time of index. In addition to this, we have now added information that the random selection of the controls was performed by Statistics Sweden which is a governmental agency, and that this approach has been used in several other registry-based studies previously conducted in Sweden. (line 207-209 in the Revised Manuscript, methods section). We have also added examples of sources from previous registry-based studies performed in Sweden that have used this method previously.

Study sample size (power) was not calculated a-priori since the number of male and female IPH perpetrators in the Västra Götaland Region between 2000-2016 was fixed and not possible to modify. The same number of matched controls has been used in previous registry-based studies performed in Sweden; examples of these studies are referred to in the manuscript (line 209).

**Response to reviewer # 2**

**Reviewer’s comments:**
Thank you for the opportunity to review the paper entitled “Healthcare utilization, mental disorders and behavioural disorders among perpetrators of intimate partner homicide in 2010-2016: a registry-based case-control study from Sweden”.
This is an original research with novel results, for which I congratulate the authors. Having said this, I consider that some aspects should be revised and improved so that the article can be publishable.

**Our response**

Thank you very much for your valuable comments and suggestions for improvement of this paper. We are grateful for these inputs and we hope that we have managed to clarify any doubts or unanswered questions with our responses, comments and changes in this paper. Our answers are found below, after each reviewer comment.

**Reviewer’s comments:
*1) Introduction:***
- The differential explanation between IPH committed by men and by women should have a somewhat broader theoretical substrate focused on the aim of the study.

**Our comments**

We have added more theoretical substrate according to your suggestions, explaining the differences between male and female perpetrated IPH in the introduction section, line 75-76, line 92-101 and line 101-110 in the revised manuscript. Further we have added more theory about female IPH and mental health problems in the introduction section, line 118-124.

**Reviewer’s comments:**
- The introduction is mainly focused on mental health problems, could be said something more specific about the contact with the health care system in order to justify the aims of the study?

**Our comments**
We have added more information about contact with the healthcare system under the introduction section, line 90-110. Most of this information overlaps with theory provided for the previous point above, i.e. male perpetrators may contact healthcare services, primarily for other reasons than mental health issues whereas female perpetrators may contact healthcare services for being exposed to IPV. Since both male and female perpetrators may contact healthcare services for a range of health issues other than mental health problems, we believe that it is important to examine general healthcare utilization of IPH perpetrators.

**Reviewer’s comments:**
- In relation to the above, why so much theoretical background on the differences between IPH perpetrators and non-lethal IPV or other non-partner victims (focused on mental health problems) to conclude that it is important to study the differences between IPH perpetrators and the general population? Why is this difference important and for what?

**Our comments**
We wanted to provide a clear outline of the findings from previous research that compares perpetrators of IPH with perpetrators of IPV and other types of homicides. This, since we wanted to highlight the differences and similarities of previous results based on comparisons between IPH perpetrators and other types of perpetrators without simplifying too much. In addition, and to our knowledge, only one previous study has compared IPH perpetrators with controls from the general population (this is mentioned in the introduction) and therefore we could not add much of previous findings based on such a comparison. Social and mental health issues may be overrepresented among homicide perpetrators and when perpetrators of IPH are compared with other types of perpetrators, they seem to be more conventional in many aspects. We wanted to add new and more knowledge to the picture by examining the differences between perpetrators and the general population without any known history of IPH. In line with this, we have added more information in the introduction section, line 159-165.

**Reviewer’s comments:
*2) Methods:***
- Given the wide variety of disorders included in the ICD-10 classification, it would be interesting to know which are the specific diagnoses (at least the most frequent), as well as the number of diagnoses of each participant and the number of times they use health care. This would lead to a clearer identification of the differences with the general population in order to make a proposal for a practical application of the results.

**Our comments:**
From previous research performed by Belfrage & Rying (2004), Lysell et al. (2016) and Caman et al. (2016), we already got valuable knowledge about specific psychiatric diagnosis among IPH perpetrators in Sweden. We wanted to add new knowledge by investigating whether IPH perpetrators in the Region of Västra Götaland, had more registered psychiatric diagnoses, regardless of specific psychiatric diagnoses, compared to individuals from the general population. We have added information in the introduction section (line 126-130) where we describe that we want to study mental and behavioural disorders from a broader perspective. If we would have focused on specific diagnoses only, we may have missed valuable information about potential differences between IPH perpetrators and the general population, as well as information about perpetrator’s general mental health diagnoses.

**Reviewer’s comments:**
- In the same way, when the health care contact is for reasons other than mental-health reasons, it would be advisable to identify those reasons.

**Our comments:**
In line with our answer in the previous point we wanted to examine health care utilization from a broader perspective. This since IPH perpetrators may seek care for a range of different reasons other than mental health problems. Our aim was to examine whether IPH perpetrators and individuals from the general population differed in healthcare contacts regardless of diagnosis. One reason for this is that a majority of IPH perpetrators are men and previous research indicates that societal norms may lead men to seek care primarily for reasons other than specific mental health concerns We have added information about this in the introduction, line 92-101.

**Reviewer’s comments:**
It would be necessary to clearly define what is meant by “social benefits”. Are they only economic benefits or other types of services?

**Our comments**
We have clarified what is included in “social benefits”(methods section, line 273-274).

**Reviewer’s comments:**
***3) Discussion and conclusions:***
- The results are really interesting as they support the evidence about a higher use of health care resources of IPH, although the percentage is low. But I consider that the reflection on their implications and their practical application in the healthcare field should be much more profound.
o Mental health diagnoses and physical health diagnoses would be comparable?

**Our comments:**
We may have misunderstood the question, but we do not believe that mental health and physical health diagnoses are comparable, and our aim has not been to compare mental health diagnoses with physical disorders. We hope that our previous answers and comments have clarified any ambiguities or misunderstandings. If not, we will gladly clarify further doubts.

**Reviewer’s comments:**
o What can be the real role of healthcare professionals in the detection of potential homicides?
o How are they going to identify or screen them?
o Have those professionals previously identified IPV in order to identify a risk of homicide?

**Our comments:**
From our perspective (which also is the recommendation from the National Board on Health and Welfare), healthcare providers need to inquire patients about IPV in order to prevent IPH. Please see or response (added text) in the revised manuscript under the discussion section, line 405-409.

**Reviewer’s comments:**
o What would be the ethical and legal implications of this?

**Our comments:**
Questions about IPV perpetration needs to be asked in a non-judgemental way and involving follow-up visits, clear communication with the patient about potential implications of the disclosure and clear routines and procedures in case of disclosure. This is added under the discussions section, 421-435. From an ethical perspective where lives may be in danger; If healthcare providers suspect hat perpetrators may be dangerous for a current or former partner, the recommendation is that healthcare providers inform the police. This is also added in the discussion section (line 434-435).

**Reviewer’s comments:**
o Would it be advisable to being coordinated with other agents, such as police, justice, social services…

**Our comments:**
Yes, we have added information about this (line 431-435).
