# Supplementary material for: Health care utilization, mental disorders and behavioural disorders among perpetrators of intimate partner homicide in 2000–2016: A registry-based case-control study from Sweden
Source: PLoS One. 2024 Feb 23;19(2):e0298693. doi: 10.1371/journal.pone.0298693 (PMC10889610; doi:10.1371/journal.pone.0298693)
Supplement: Attachment — Submitted filename: Response to reviewers.docx [file pone.0298693.s002.docx]

**Response to reviewers**

**Journal Requirements:**

Please review your reference list to ensure that it is complete and correct. If you have cited papers that have been retracted, please include the rationale for doing so in the manuscript text, or remove these references and replace them with relevant current references. Any changes to the reference list should be mentioned in the rebuttal letter that accompanies your revised manuscript. If you need to cite a retracted article, indicate the article’s retracted status in the References list and also include a citation and full reference for the retraction notice.

**Our comments:**
We have checked our reference list and ensured that it is complete and correct; there are no retracted papers included. In the previous revision and after suggestions from reviewers, we added some clarifications and more theory. Thus, the references that we added to the previous revision (submitted in December 2023) are the following references:

- Voce I, Bricknell S. Female perpetrated intimate partner homicide: Indigenous and non-Indigenous offenders: Australian Institute of Criminology; 2020.
- Vickery A. Men's Help-Seeking for Distress: Navigating Varied Pathways and Practices. Frontiers in sociology. 2021;6:724843-. doi: 10.3389/fsoc.2021.724843.
- Bonomi AE, Anderson ML, Rivara FP, Thompson RS. Health Care Utilization and Costs Associated with Physical and Nonphysical-Only Intimate Partner Violence. Health services research. 2009;44(3):1052-67. doi: 10.1111/j.1475-6773.2009.00955.x.
- Petersen R, Gazmararian J, Andersen Clark K. Partner violence: implications for health and community settings. Women's health issues. 2001;11(2):116-25. doi: 10.1016/S1049-3867(00)00093-1.
- Shilan C, Joakim S, Katarina H. Mental Disorders and Intimate Partner Femicide: Clinical Characteristics in Perpetrators of Intimate Partner Femicide and Male-to-Male Homicide. Frontiers in psychiatry. 2022;13. doi: 10.3389/fpsyt.2022.844807.
- Weizmann-Henelius G, Matti Grönroos L, Putkonen H, Eronen M, Lindberg N, Häkkänen-Nyholm H. Gender-Specific Risk Factors for Intimate Partner Homicide: A Nationwide Register-Based Study. Journal of interpersonal violence. 2012;27(8):1519-39. doi: 10.1177/0886260511425793.
- Ludvigsson J, Almqvist C, Bonamy A-K, Ljung R, Michaëlsson K, Neovius M, et al. Registers of the Swedish total population and their use in medical research. European Journal of Epidemiology. 2016;31(2):125-36. doi: 10.1007/s10654-016-0117-y
  .
- Fazel S, Lichtenstein P, Grann M, Långström N. Risk of violent crime in individuals with epilepsy and traumatic brain injury: a 35-year Swedish population study. PLoS medicine. 2011;8(12):e1001150-e. doi: 10.1371/journal.pmed.1001150.
- Lysell H, Runeson B, Lichtenstein P, Långström N. Risk factors for filicide and homicide: 36-year national matched cohort study. The journal of clinical psychiatry. 2014;75(2):127-32. doi: 10.4088/JCP.13m08372.
- Salinas Fredricson A, Naimi-Akbar A, Adami J, Lund B, Rosén A, Hedenberg-Magnusson B, et al. Diseases of the musculoskeletal system and connective tissue in relation to temporomandibular disorders—A SWEREG-TMD nationwide case-control study. PloS one. 2022;17(10):e0275930-e. doi: 10.1371/journal.pone.0275930.
- Lysell H, Dahlin M, Viktorin A, Ljungberg E, D'Onofrio BM, Dickman P, et al. Maternal suicide - Register based study of all suicides occurring after delivery in Sweden 1974-2009. PloS one. 2018;13(1):e0190133-e. doi: 10.1371/journal.pone.0190133.
- Michau LMA, Horn JM, Bank ABA, Dutt MJD, Zimmerman CP. Prevention of violence against women and girls: lessons from practice. The Lancet (British edition). 2015;385(9978):1672-84. doi: 10.1016/S0140-6736(14)61797-9.
- Gerlock AA, Grimesey JL, Pisciotta AK, Harel O. Documentation of screening for perpetration of intimate partner violence in male veterans with PTSD. The American journal of nursing. 2011;111(11):26. doi: 10.1097/01.NAJ.0000407296.10524.d7.
- Kimberg LS. Addressing Intimate Partner Violence with Male Patients: A Review and Introduction of Pilot Guidelines. Journal of general internal medicine : JGIM. 2008;23(12):2071-8. doi: 10.1007/s11606-008-0755-1.
- Portnoy GA, Colon R, Gross GM, Adams LJ, Bastian LA, Iverson KM. Patient and provider barriers, facilitators, and implementation preferences of intimate partner violence perpetration screening. BMC health services research. 2020;20(1):746-. doi: 10.1186/s12913-020-05595-7.
- Taket A, Nurse J, Smith K, Watson J, Shakespeare J, Lavis V, et al. Routinely asking women about domestic violence in health settings. BMJ. 2003;327(7416):673-6. doi: 10.1136/bmj.327.7416.673.
- The National Board of Health and Welfare. Våld i nära relationer- Handbok för socialtjänsten , hälso och sjukvården och tandvården (Domestic violence – A handbook for social services, health care and dental care). 2023.

**Additional Editor Comments:**

Dear authors, on this occasion, one of the reviewers was unable to assess the changes made. Based on my own judgement, I believe that the manuscript has been substantially improved. There is only one final contribution left from reviewer 2. Please evaluate this.
Congratulations on the work you have done.

**Our comments:**

Thank you for this positive comment and news. We have made suggestions according to the reviewer’s comment/suggestion. Please see our answer below.

**Reviewer #2:**
I reiterate my congratulations to the authors for their work. The responses to the comments are very pertinent and the manuscript has improved. I would like to make one last suggestion for change before the document is finally accepted:

In the Discussion, line 446, reads "...concerns for the health and well-being of both the perpetrator and others are expressed [47]". I think that this "others" should make visible and expressly refer (in the way the authors consider most appropriate) to the people who are affected by this violence, especially the direct victims.

**Our comment:**

First of all, we are extremely grateful for all the help we have received in improving the content of this manuscript. According to your suggestion, in line 426-428 (in the revised manuscript) we have changed/added the text as follows: *“Furthermore, it is important to address IPV as a healthcare issue, expressing concerns for the health and well-being of both the perpetrator, the victim, and their children.”* We hope that this has clarified the content of the sentence.
